# P211: Organization and scope of infection control in Polish hospitals. First results of the pan-european prohibit survey

**DOI:** 10.1186/2047-2994-2-S1-P211

**Published:** 2013-06-20

**Authors:** A Różańska, J Wójkowska-Mach, PB Heczko, M Bulanda

**Affiliations:** 1 Chair of Microbiology, Jagiellonian University Medical College, Kraków, Poland

## Introduction

The PROHIBIT "Prevention of Hospital Infections by Intervention and Training" survey was initiated to obtain data on practices on HAI prevention and to identify enabling factors or barriers to compliance with evidence-based recommendations.

## Objectives

The paper presents initial descriptive results of a survey on organization of surveillance programs in Polish hospitals, which is a part of PROHIBIT project.

## Methods

Survey was performed by means of the standardized questionnaire in the year 2012. Questions were answered by IC personnel.

Completed questionnaires were obtained from 9 hospitals of different size and type.

## Results

Infection control team (ICT) works in every hospital and the head of the team in 8 hospitals is a physician. In most hospitals number of epidemiological nurses per 100 beds ranges from 0.4 to 0.8.

In every hospital surveillance comprises all the most important from the epidemiological point of view forms of infections: SSI, BSI, PNEU, UTI, CDI and MDRO - in all wards.

Infection cases in 5 hospitals are documented by epidemiological nurse in collaboration with infection control physician or physician of the ward. In rest - by infection control physician.

Most hospitals (7 of 9) also declare running the post-discharge surveillance of SSI.

Feedback on infection rates to HCWs are given twice a year in most hospitals.

In 7 of 9 hospitals consumption of alcohol-based handrub are monitored(average usage is about 10 l /100 admissions per year). ICTs in all hospitals have access to microbiological data. Average number of blood cultures per 100 admissions/year is 14.8.

## Conclusion

The results obtained from this small group may suggest that the surveillance programs are complex and well organized.

But, this kind of process measure data are insufficient for assessing the safety of hospitalization which demands the outcomes measure data which are not available in Poland presently. There are just single publications presenting the epidemiological data on infections in Polish hospitals in professional literature what with the low percentage of completed questionnaires suggests the infection control in Poland requires varied educational and practical activities in order to improve safety of hospitalization.

## Disclosure of interest

None declared

